# LncRNA TP73-AS1 promoted the progression of lung adenocarcinoma via PI3K/AKT pathway

**DOI:** 10.1042/BSR20180999

**Published:** 2019-01-11

**Authors:** Chunfeng Liu, Lei Ren, Jun Deng, Songping Wang

**Affiliations:** 1 Respiratory Medicine Department 1, The Affiliated Hospital of Southwest Medical University, Luzhou 646000, Sichuan, China; 2 Gastroenterological Surgery, The Affiliated Hospital of Southwest Medical University, Luzhou 646000, Sichuan, China

**Keywords:** growth, lung adenocarcinoma, metastasis, PI3K/AKT, TP73-AS1

## Abstract

Lung adenocarcinoma (LAD) is one of the most common malignancies that threats human health worldwide. Long non-coding RNAs (lncRNAs) have been reported to play significant roles in tumorigenesis and might be novel biomarkers and targets for diagnosis and treatment of cancers. TP73-AS1 is a newly discovered lncRNA involved in the tumorigenesis and development of several cancers. However, its role in LAD has not been investigated yet. In the present study, we first found that TP73-AS1 expression was markedly increased in LAD tissues and cell lines and its overexpression was strongly associated with poor clinical outcomes. Then the loss/gain-of-function assays elucidated that TP73-AS1 contributed to cell proliferation, migration, and invasion *in vitro*, and the *in vivo* experiments illustrated that its knockdown inhibited tumor growth and metastasis. What was more, we discovered that phosphoinositide 3-kinase and AKT (PI3K/AKT) pathway was activated both in LAD tissues and cell lines but inactivated under TP73-AS1 silence. Moreover, the activation of this pathway could rescue the inhibitory effects of TP73-AS1 suppression on LAD cellular processes partially. These data suggested that TP73-AS1 served as an oncogene in LAD partially through activating PI3K/AKT pathway and it could be a potential target for diagnosis and treatment of LAD.

## Introduction

Lung cancer is one of the most common primary malignancies and the primary cause of cancer-related deaths worldwide [1]. Lung adenocarcinoma (LAD), the most common subtype of non-small-cell lung cancer (NSCLC) which accounts for the majority of all primary lung cancer cases diagnosed, has a low 5-year survival rate [2,3]. In the past decades, we have made great improvements in the treatment of NSCLC, such as anti-PD-1/PD-L1 therapy [4,5], targetted therapies [6,7], which are also suitable for LAD treatment [8,9]. Meanwhile, the therapeutic strategies for treating LAD also improved [10–12]. Nevertheless, patients with LAD still did not survive. In this regard, it is necessary to find out more sensitive targets for LAD treatment.

Long non-coding RNAs (lncRNAs), a class of transcripts that lack ORF, have more than 200 bases in length [13,14]. It has been verified over the years that lncRNAs are involved in various cellular processes, tumorigenesis, and the development of various human cancers [15–18]. In addition, a large number of lncRNAs have been demonstrated to play important roles in lung cancer [19–21], even in LAD [22–25].

Increasing evidence showed that TP73-AS1 (P73 antisense RNA 1T), an lncRNA located on chromosome 1p36, is implicated in the progression of several cancers. For example, knockdown of lncRNA TP73-AS1 inhibits cell proliferation and induces apoptosis in esophageal squamous cell carcinoma [26]. The lncRNA TP73-AS1 modulates HCC cell proliferation through miR-200a-dependent HMGB1/RAGE regulation [27]. And TP73-AS1 also acts as an oncogene in promoting cell proliferation, invasion, and migration in breast cancer via different regulating loops [28,29]. Interestingly, despite its discovered oncogenic role in multiple cancers, TP73-AS1 can also serve as a tumor suppressor in bladder cancer via EMT pathway [30], and it has been identified to act as tumor suppressor via sponging human-specific miR-941 [31]. However, its role and function in LAD remains unknown.

Phosphoinositide 3-kinase and AKT (PI3K/AKT) pathway has been identified as one of the ten classic oncogenic signaling pathways, and the genetic alterations in this pathway always lead to aberrant cell growth in cancers [32]. In addition, cellular processes could also be influenced by its downstream targets, which initiate and regulate development of cancers [33]. Activation of this signaling pathway finally results in suppression of cell autophagy and enhancement of development in cancer [34].

In the present study, we aimed to identify the function and potential mechanism of TP73-AS1 in LAD progression.

## Materials and methods

### Clinical samples

A total of 80 specimens were obtained from The Affiliated Hospital of Southwest Medical University. The clinical data of these specimens, such as age, gender, smoking, tumor size, differentiation, lymph node metastasis and TNM stage, were collected here. Small pieces of the specimens were maintained in the liquid nitrogen for the following experiments. The Ethics Committee of Southwest Medical University (registration number: 2017AHSMU-048) approved our study. Every patient had received no other therapies prior to operation and signed the informed consent before the study. The experiments with human specimens were conducted in the light of the World Medical Association Declaration of Helsinki.

### Cell lines and culture

Normal human bronchial epithelial cells (BEAS-2B) and LAD cell lines (H157, A549, H1299, H1975, and HCC827) were obtained from ATCC (Manassas, VA, U.S.A.) and grown in RPMI 1640 with 10% FBS (Gibco®, Waltham, MA, U.S.A.) and penicillin/streptomycin in a humid atmosphere with 5% CO_2_ at 37°C.

### Cell transfection

shRNAs specifically against TP73-AS1 and corresponding control were obtained from Santa Cruz Biotechnology Inc. (Dallas, TX, U.S.A.) and then subcloned into PX459 plasmid (Plasmid #48139; Addgene, China). Negative vector transfected cells were used as a negative control (shCtrl). After transfection for 72 h, puromycin with the concentration of 1.5 μg/ml was added to select the stably transfected cells. The medium with puromycin was replaced every 3 days and the cell selection continued for ∼3 weeks. Thereafter, the chosen cells were maintained at 37°C in a humid atmosphere with 5% CO_2_ for use in the following experiments.

### RNA isolation and q-RT-PCR

TRIzol reagent (Invitrogen) was utilized to separate RNAs from tissues and cells. Then total RNA was transcribed reversely to cDNA using PrimeScript RT Reagent Kit (Takara, Dalian, China). Based on the user guide, real-time PCR analysis was carried out by SYBR Premix Ex Taq (Takara). GAPDH acted as the internal control. Experiments were conducted in triplicates. The primer sequences of TP73-AS1 and GAPDH (normalized control) were listed: TP73-AS1 forward: 5′-CCGGTTTTC CAGTTCTTGCAC-3′ and reverse: 5′-GCCTCACAGGGA AACTTCATGC-3′; GAPDH forward: 5′-AGAAGGCTGGGGCTCATTTG-3′ and reverse: 5′-AGGGGCCATCCACAGTCTTC-3′ [30]; β-actin forward: 5′-GCACCACACCTTCTACAATG-3′ and reverse: 5′-TGCTTGCTGATCCACATCTG-3′.

### MTT assay

First of all, cells (with a concentration of 3 × 10^3^ cells/well) from different groups were plated into 96-well plates and cultured for 4 h. After that, cells were further cultured for respective 0, 24, 48, 72, and 96 h. Then 20 μl MTT solution was added into cells after incubation followed by the addition of DMSO. Finally, FlexStation 3 plate reader (Molecular Devices, CA) was used to determine the amount of viable cells by examining the absorbance at 490 nm. The experiment was performed for three times.

### Colony formation assay

After transfection with shCtrl or shTP73-AS1, cell suspensions cultured in RPMI 1640 were added into six-well plates at a density of 1 × 10^3^ cells/well. The plates were then maintained under a humidified atmosphere containing 5% CO_2_ at 37°C. After incubation for 2 weeks, the dishes were fixed using methanol and stained with 0.1% Crystal Violet, followed by manually counting the number of colonies with more than 50 cells.

### Flow cytometry assay

To analyze cell cycle progression of transfected LAD cells, propidium iodide cell cycle detected kits (BD, U.S.A.) were applied under the manufacturer’s directions. And cell apoptosis was determined by Annexin V-FITC apoptosis detection kits (Selleck, Shanghai, China) on the basis of the manufacturer’s instructions. The results were evaluated using flow cytometry (FACSCalibur, BD Biosciences). The experiments were carried out in triplicates.

### Transwell assays

Cell migration and invasion ability *in vitro* was determined using transwell assay. For the migration assay, the suspension of LAD cells (1 × 10^5^) transfected with either shRNA targetting TP73-AS1 or shCtrl were added into the upper chamber of the transwell, and RPMI 1640 containing 10% FBS was added into lower chamber of the transwell. After 24-h incubation, cells migrating to the lower surface were fixed using 4% PFA and then stained using 0.3% Crystal Violet before being calculated by microscope. For the invasion assay, the inserts in the incubator were pre-coated by 50 μl of matrigel solution (1:4 mixed with PBS; BD, Franklin Lakes, NJ, U.S.A.) at 37°C for 2 h, and the following steps were performed as the migration procedure except the number of cells used in invasion assay was 2 × 10^5^ cells/well. Experiments were independently performed for three times.

### *In vivo* animal studies

Five-week-old male BALB/c nude mice obtained from the Slac Laboratory Animal Center (Shanghai, China) were raised and maintained on the basis of the institutional policies. All animal experiments were carried out following the experimental animal use guidelines of the National Institutes of Health. For the *in vivo* tumorigenesis experiments, shTP73-AS1 or shCtrl stably transfected A549 and HCC827 cells were harvested and injected into the left flanks of mice subcutaneously (2 × 10^6^ cells/mice, *n*=8 for each group). The tumor volume was measured every 4 days and then calculated according to the following formula: V = 0.5 × D × d^2^ (V means volume, D refers to longitudinal diameter, and d indicates latitudinal diameter). Then the mice were killed and the tumors were gathered 5 weeks later. For the metastasis evaluation, the mice were intravenously injected with 4 × 10^6^ cells suspended in 400 μl PBS and then killed after 4 weeks of injection. Afterward, the tumors in livers were observed and counted. All procedures received the approval of the Animal Use and Care Committee of the Southwest Medical University.

### Immunohistochemistry

For immunohistochemical analyses, tissues from two groups of mice were embedded in paraffin, chopped into 4-μm-thick sections and then fixed with 4% paraformaldehyde. The sections were next incubated with primary monoclonal antibody against Ki-67 (#9449, Cell Signaling Technology, Danvers, MA, U.S.A.). Afterward, the sections were further incubated for 30 min at room temperature with horseradish peroxidase–conjugated IgG which served as secondary antibody, followed by incubation with 3,3′-Diaminobenzidine (3,3′-DAB, Maxim, Fuzhou, China) for 5 min. After counterstaining with Hematoxylin for 30 s, the sections were photographed under a TE2000 microscope (Nikon, Tokyo, Japan).

### Western blot

Proteins from tissues or cells were extracted using RIPA buffer containing protease and phosphatase inhibitors. The quantities of proteins extracted from different cell dissolutions were determined by the BCA protein assay kit (Thermo Fisher, U.S.A.) before using. After that, proteins were separated electrophoretically under SDS/PAGE and then shifted to the NC membrane. Then the membrane was blocked by 10% non-fat milk at room temperature for 1 h. After washing with TBS and Tween 20 (TBST), the primary antibodies were added respectively. After incubating overnight, the membranes were washed by TBST for three times. And then the corresponding secondary antibody (diluted to 1:2000) was applied and incubated for 1 h at 37°C, and then the membrane was washed with TBST for three times. Signal visualization was performed by ECL substrates (Millipore, MA, U.S.A.), and GAPDH was the normalized control. The gray intensity was estimated by ImageJ software (NIH). Primary antibodies used in the present study were listed as follows: anti-PI3K (#4249), anti-p-PI3K (#4228), anti-AKT (#9272), anti-p-AKT (#4060), anti-MEK1/2 (#4694), anti-p-MEK1/2 (#2338), anti-cleaved caspase 3 (#9661), anti-GAPDH (#5174) (all above from CST Company, U.S.A.); anti-caspase 3 (ab13585), anti-Bax (ab32503), anti-PUMA (ab9643), anti-β actin (ab8226) (these three antibodies from Abcam, Cambridge, U.K.).

### Statistical analysis

All the data obtained from three independent experiments at least were assessed using GraphPad Prism 5.0 (GraphPad Software, La Jolla, CA, U.S.A.) and showed as mean ± S.D. Differences between two groups were determined by Student’s *t*test and those amongst more than two groups were analyzed using ANOVA with post-test. The Kaplan–Meier analysis and log-rank test were performed to evaluate overall survival (OS). And the intensity of Western blots signal was quantitated by ImageJ. Statistical significances were defined as *P*<0.05.

## Results

### Expression of TP73-AS1 was up-regulated in LAD tissues and cell lines

**Figure 1:**
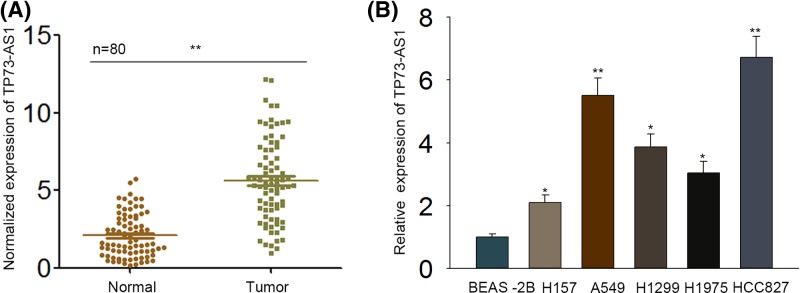
Expression of TP73-AS1 was up-regulated in LAD tissues and cell lines The expression of TP73-AS1 was estimated by qRT-PCR. (**A**) TP73-AS1 expression was significantly increased in 80 paired LAD tissues compared with adjacent non-tumor tissues. (**B**) TP73-AS1 was expressed highly in LAD cell lines compared with that in normal human bronchial epithelial cells (BEAS-2B), and its expression in A549 and HCC827 cells was higher than that of any other detected cancer cell lines. The results above were obtained from three independent experiments at least. *P<0.05, **P<0.01.

To explore whether TP73-AS1 impacted LAD or not, we examined TP73-AS1 expression in LAD tissues first. The expression levels of TP73-AS1 in tumor tissues and corresponding non-tumor tissues from 80 patients with LAD were detected by qRT-PCR. In result, TP73-AS1 expression in LAD tissues was extremely high than that in corresponding non-tumor tissues (Figure 1A). Besides, the levels of TP73-AS1 expression were also detected in LAD cell lines (H157, A549, H1299, H1975, and HCC827) and normal human bronchial epithelial cells (BEAS-2B), which were also discovered to be obviously increased in all detected LAD cells compared with BEAS-2B cells (Figure 1B). In addition, the expression of TP73-AS1 was higher in A549 and HCC827 cells than any other cells examined here.

### Expression levels of TP73-AS1 were closely associated with poor prognosis in patients with LAD

**Figure 2:**
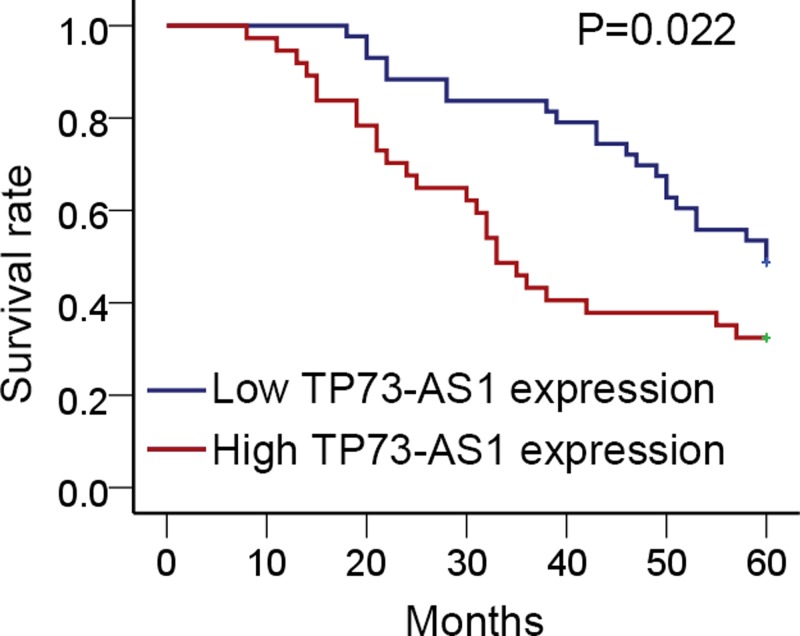
Expression of TP73-AS1 was related to poor survival in patients with LAD The association between TP73-AS1 expression and prognosis of patients with LAD was detected by Kaplan–Meier analysis and the log-rank test. The prognosis of LAD patients was evaluated by OS. A total of 80 LAD patients were involved in the present study, amongst which 43 were with low TP73-AS1 level and 37 with high TP73-AS1 level.

To make sure whether the expression of TP73-AS1 was related to clinical and pathological features as well as the prognosis of patients with LAD, the correlation between clinicopathological parameters and TP73-AS1 expression levels was analyzed. A total of 80 LAD patients were stratified by high (*n*=37) or low (*n*=43) TP73-AS1 level according to the median value. It was indicated that TP73-AS1 expression levels were significantly associated with TNM stage and lymph node metastasis but had no concern with other clinical characteristics (Table 1). In addition, proportional hazards method analysis showed that TP73-AS1 expression could be of independent prognostic importance in LAD, similar to tumor size, TNM stage, and lymph node metastasis (Table 2). Moreover, patients with high TP73-AS1 expression always suffered from a poorer OS than those with low levels of TP73-AS1 (Figure 2). These results revealed that TP73-AS1 might act as a biomarker for prognosis of LAD.

**Table 1:** Relationships between expression levels of TP73-AS1 and clinicopathological characteristics of patients with LAD

Variable	TP73-AS1 expression		*P*-value
	Low	High	
**Age**			
>65	8	5	0.406
≤65	35	32	
**Gender**			
Male	28	29	0.724
Female	15	8	
**Smoking**			
Yes	13	8	0.412
No	30	29	
**Tumor Size**			
≤5	30	11	0.094
>5	13	26	
**Differentiation**			
Well	1	4	0.859
Moderate-Poor	42	33	
**Lymph node metastasis**			
Negative	28	10	0.043
Positive	15	27	
**TNM stage**			
I + II	32	13	0.014
III	11	24	

Low/high by the sample median. Pearson χ^2^ test. *P*<0.05 was considered statistically significant. Data were obtained from 80 LAD patients who had undergone operations in The Affiliated Hospital of Southwest Medical University.

**Table 2:** Multivariate analysis of prognostic parameters in patients with LAD by Cox regression analysis

Variable	Category	*P*-value
**Age**		
	>65	0.762
	≤65	
**Gender**		
	Male	0.223
	Female	
**Smoking**		
	Yes	0.450
	No	
**Tumor size**		
	≤5	0.001*
	>5	
**Differentiation**		
	Well	0.176
	Moderate-Poor	
**Lymph node metastasis**		
	Negative	0.001*
	Positive	
**TNM stage**		
	I + II	0.001*
	III	
**TP73-AS1 level**		
	High	0.005*
	Low	

Proportional hazards method analysis showed a positive, independent prognostic importance of TP73-AS1 expression (*P*=0.005). *P<0.05 was considered statistically significant. Data were obtained from 80 LAD patients who had undergone operations in The Affiliated Hospital of Southwest Medical University.

### TP73-AS1 promoted cell proliferation but repressed apoptosis *in vitro*

**Figure 3:**
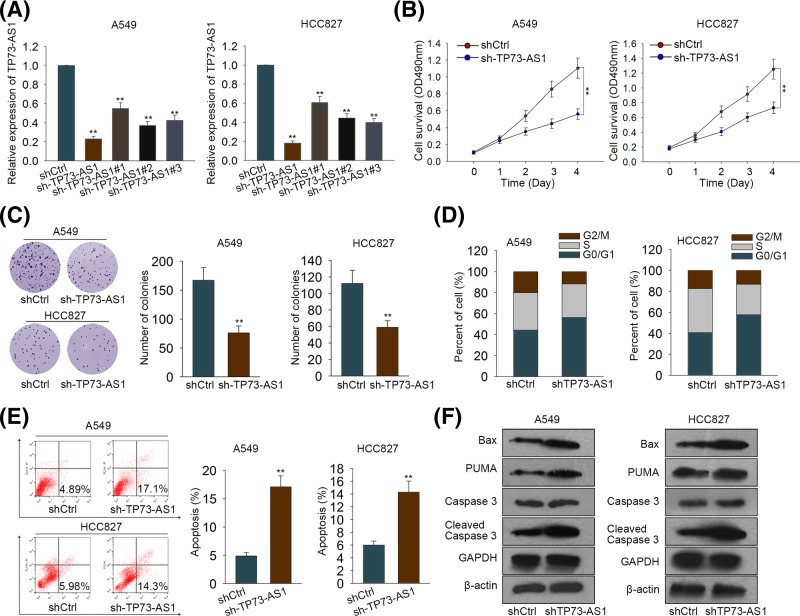
Knockdown of TP73-AS1 attenuated cell proliferation but induced apoptosis in LAD cells (**A**) qRT-PCR was used to analyze the transfection efficiency of shTP73-AS1 in A549 and HCC827 cells. Cells transfected with silencer negative control shRNA acted as negative control (shCtrl). (**B**) MTT assay was conducted to evaluate the impact of TP73-AS1 on cell survival. (**C**) Colony formation assay was utilized to examine the function of TP73-AS1 in colony formation ability of A549 and HCC827 cells. (**D**,**E**) Flow cytometry analysis was utilized to determine the cell cycle distribution and the apoptosis rate of A549 and HCC827 cells. (**F**) The protein levels of apoptosis-related genes were tested by Western blotting. All these assays were conducted triply. **P<0.01.

To discover the effects of TP73-AS1 on LAD, we next down-regulated the expression of TP73-AS1 in A549 and HCC827 cells. And cells transfected with negative vector shRNA served as the negative control (shCtrl). The expression levels of TP73-AS1 were remarkably decreased in A549 and HCC827 cells after transfection with four different kinds of shTP73-AS1 compared with the cells transfected with shCtrl (Figure 3A). Due to the optimum silence efficiency amongst the four, shTP73-AS1 was chosen for the subsequent experiments. The results of CCK8 assay demonstrated that knockdown of TP73-AS1 remarkably decreased the cell survival rate in A549 and HCC827 cells (Figure 3B). Moreover, knockdown of TP73-AS1 showed a marked reduction in colony formation ability in either A549 or HCC827 cells (Figure 3C). Additionally, silenced TP73-AS1 led to G_0_/G_1_ cell cycle arrest, whereas induced apoptosis meanwhile in both A549 and HCC827 cells (Figure 3D,E). Consistently, pro-apoptosis proteins, such as Bax, PUMA, and cleaved caspase 3, were increased after silencing TP73-AS1 in LAD cells (Figure 3F). However, overexpression of TP73-AS1 in BEAS-2B cells exhibited elevated cell proliferation ability and reduced cell apoptosis rate (Supplementary Figure S1A–E). These results indicated that TP73-AS1 promoted cell proliferation while reduced apoptosis in LAD cells.

### TP73-AS1 facilitated cell migration and invasion in LAD cells

**Figure 4:**
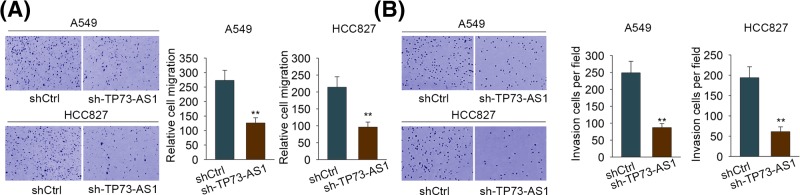
Knockdown of TP73-AS1 induced suppressed cell migration and invasion in LAD cells (**A**,**B**) Cell migration and invasion abilities were respectively examined by transwell migration and invasion assay both in A549 and HCC827 cells. These assays were repeated three times. **P<0.01.

Since the expression level of TP73-AS1 was significantly correlated with lymph node metastasis (Tables 1 and 2), we suspected that TP73-AS1 also played a role in tumor metastasis in LAD. To confirm the role of TP73-AS1 in tumor metastasis of LAD, we first investigated whether TP73-AS1 affected cell migration and invasion *in vitro*. As displayed in Figure 4A, the results of Transwell migration assays suggested significant decreases in the migration abilities in the shTP73-AS1-transfected group in comparison with the shCtrl-transfected group in either A549 or HCC827 cells. Similarly, the capacities of cell invasion were also evidently repressed under TP73-AS1 inhibition (Figure 4B). In a word, TP73-AS1 knockdown remarkably repressed cell migration and invasion in LAD cells. In contrast, TP73-AS1 overexpression increased the number of migrated or invaded cells in BEAS-2B cells, namely its overexpression accelerated BEAS-2B cell migration and invasion (Supplementary Figure S1F). Taken together, TP73-AS1 played a role in promoting cell migration and invasion in LAD cells.

### Silencing TP73-AS1 suppressed tumor growth and metastasis *in vivo*

**Figure 5:**
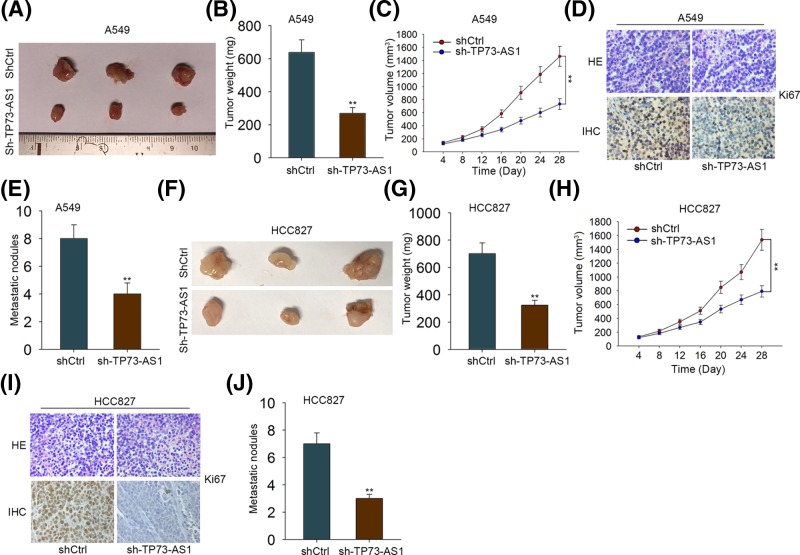
Silencing TP73-AS1 suppressed tumor growth and metastasis *in vivo* (**A**) Images of tumors derived from mice with the injection of shCtrl or shTP73-AS1 transfected A549 cells. (**B**,**C**) Tumor weights and tumor growth curves of mice injected with shCtrl or shTP73-AS1 transfected A549 cells. (**D**) Ki-67 staining in tumors derived from shCtrl or shTP73-AS1 transfected A549 cells was assessed using HE and immunohistochemical staining. Scale bar: 100 μm. (**E**) The number of liver metastatic nodules in mice with the injection of shCtrl or shTP73-AS1 transfected A549 cells was counted. (**F**–**J**) The results of *in vivo* experiments performed by using shCtrl or shTP73-AS1 transfected HCC827 cells were obtained as above. Images of tumors (F), tumor weights (G), tumor growth curves (H), Ki-67 staining (I), and liver metastatic nodules (J). **P<0.01.

To further probe the significance of TP73-AS1 in tumor growth and metastasis, TP73-AS1 silenced A549 and HCC827 cells were subcutaneously introduced into the left flanks of nude mice. Seen from Figure 5A, the mean tumor size in mice transfected with TP73-AS1 silenced A549 cells was markedly smaller than that in control mice. Correspondingly, the mean tumor weight derived from shTP73-AS1 transfected A549 cells was strikingly lower than that of tumors from shCtrl group (Figure 5B). Additionally, the tumor growth rate was significantly reduced after silencing TP73-AS1 (Figure 5C). Moreover, the tumors originating from TP73-AS1 knockdown A549 cells showed a weaker Ki-67 staining in contrast with those from the control group (Figure 5D). Furthermore, the number of liver metastatic nodules in mice injected with TP73-AS1 silenced A549 cells were distinctly less than those with shCtrl transfected cells (Figure 5E). Consistently, it was illustrated that silencing TP73-AS1 also suppressed growth and metastasis of tumors originated from HCC827 cells (Figure 5F–J). Collectively, these data revealed that TP73-AS1 contributed to LAD tumor growth and metastasis.

### TP73-AS1 promoted the progression of LAD through activating PI3K/AKT pathway

**Figure 6:**
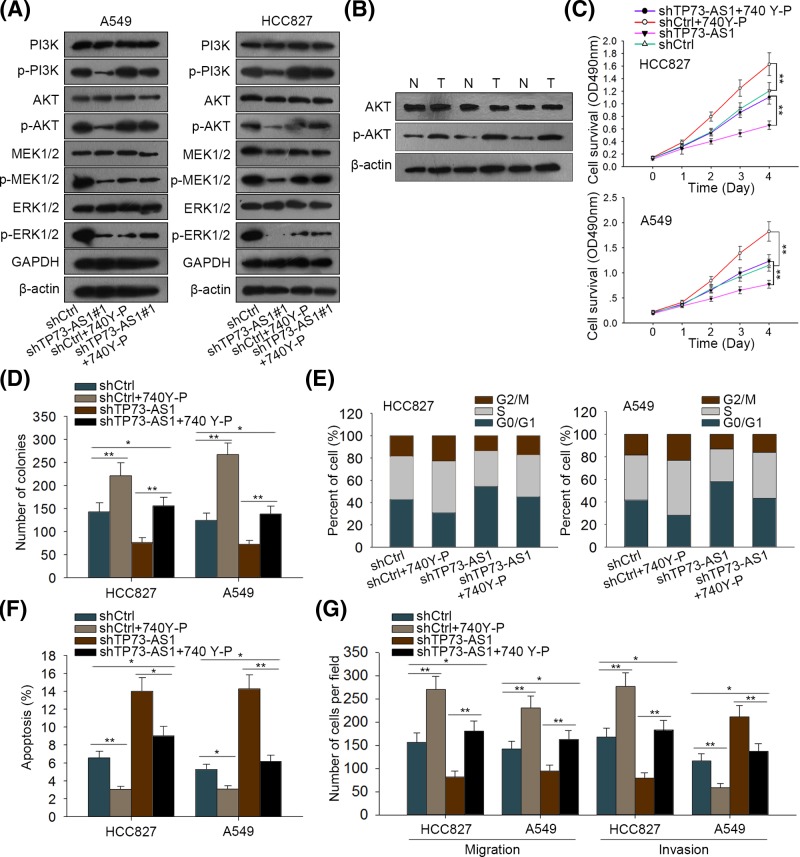
TP73-AS1 aggravated the progression of LAD via PI3K/Akt signaling pathway (**A**) The expression of proteins involved in PI3K/AKT and MRK/ERK pathway was determined by Western blotting in shCtrl or shTP73-AS1 transfected A549 and HCC827 cells after treated with or without 740Y-P, the PI3K activator. (**B**) Relative protein level of total AKT and p-AKT in LAD tissues was evaluated by Western blot assay and quantitated using ImageJ software. (**C**,**D**) MTT assay and colony formation assay were applied to detect the changes of cell proliferation ability of HCC827 and A549 cells after the transfection with shCtrl or shTP73-AS1 and the treatment with or without 740Y-P. (**E**,**F**) Cell cycle distribution and apoptosis rate altered in shCtrl or shTP73-AS1 transfected HCC827 and A549 cells after treating with or without 740Y-P was estimated by flow cytometry analysis. (**G**) Transwell assay was utilized to test the alterations of migration and invasion capacities in TP73-AS1 silenced LAD cells with or without the treatment of 740Y-P. The experiments were performed in triplicate. *P<0.05, **P<0.01.

PI3K/AKT pathway has been reported to contribute to various cancers, including LAD [35], and we supposed that TP73-AS1 might impact on LAD through this way. To validate the suspicion, Western blot assay was first utilized to detect the alterations in proteins that involved in PI3K/AKT pathway. As demonstrated in Supplementary Figure S2A, knockdown of TP73-AS1 caused a considerable reduction in the level of p-PI3K and p-AKT in both A549 and HCC827 cells, and the shTP73-AS1#1 which showed more effective silencing efficiency was chosen for following assays. Due to the importance of MEK/ERK signaling pathway in promoting cell survival, we also examined whether TP73-AS1 had an impact on this pathway and revealed that the phosphorylation level of PI3K and AKT as well as that of MEK and ERK was down-regulated under TP73-AS1 silence in the two LAD cells (Figure 6A). Based on this, we demonstrated that TP73-AS1 could stimulate the activation of both PI3K/AKT and MEK/ERK pathway in LAD cells. Nevertheless, on account of the dual functions of PI3K/AKT pathway in both cell growth as well as invasion and migration, we further focussed on this pathway here whereas the MEK/ERK pathway in the future. Furthermore, we found that the level of p-AKT was obviously up-regulated in LAD tissues and five cell lines (Figure 6B and Supplementary Figure S2B).

To further confirm the relation between PI3K/AKT activity and TP73-AS1, 740Y-P (a PI3K agonist) was applied in both HCC827 and A549 cells. First, 740Y-P was confirmed to activate PI3K/AKT pathway but not the MEK/ERK signaling (Figure 6A), and the addition of 740Y-P elevated the decreased levels of p-PI3K and p-AKT in two LAD cells transfected with shTP73-AS1#1 (Figure 6A and Supplementary Figure S2A). Then the results of MTT and colony formation assays indicated that activation of PI3K/AKT pathway caused an enhancement of cell proliferation in shTP73-AS1 group compared with the control group (Figure 6C,D). As shown in Figure 6E,F, 740Y-P attenuated G_0_/G_1_ cell cycle arrest and enhanced cell apoptosis which were induced by TP73-AS1 knockdown in two LAD cells. The results of transwell assay revealed improved capacities of cell migration and invasion upon the co-treatment with 740Y-P in TP73-AS1 silenced HCC827 and A549 cells (Figure 6G). Meanwhile, the application of 740Y-P also had effects on cell proliferation, apoptosis, migration, and invasion of HCC827 cells, suggesting that PI3K/AKT pathway could affect biological processes in LAD itself (Figure 6C–G). Taken together, TP73-AS1 elicited carcinogenesis in LAD via activating PI3K/AKT signaling pathway.

## Discussion

In the last decade, a wide range of lncRNAs have been found to be dysregulated in various human malignancies [36–39]. Recently, a growing number of reports have identified that the abnormal expression of lncRNAs is closely related to aberrant cellular processes in LAD. For example, activated lncRNA-NEAT1 expression promotes Galectin-3 activating TLR4/NF-κB signaling to promote LAD cell proliferation [40]. Overexpression of HOTTIP promotes proliferation and drug resistance of LAD by regulating AKT signaling pathway [41]. LncRNA MUC5B-AS1 promotes metastasis through mutually regulating MUC5B expression in LAD [42]. However, the functions of multiple other lncRNAs in LAD have never been identified. In the present study, we investigated the role and function of TP73-AS1 in this disease.

TP73-AS1 is a newfound lncRNA that locates on chromosome 1p36, which has been proved to be an oncogene in esophageal squamous cell carcinoma, hepatocellular carcinoma, and breast cancer [27–29,43], but it also been reported as a tumor suppressor in bladder cancer [30]. In the present study, we found that TP73-AS1 was highly expressed in LAD tissues and cell lines and its up-regulation always led to short OS time. In addition, we unveiled that TP73-AS1 promoted cell proliferation, migration, and invasion *in vitro* and its knockdown suppressed tumor growth and metastasis *in vivo*. All the data suggested that TP73-AS1 served as an oncogene in LAD.

PI3K/AKT pathway is one of the most common oncogenic signaling cascades whose aberrant regulation affects many cellular processes in a range of human cancers [44]. For instance, suppression of the PI3K/Akt signaling pathway by down-regulating monocarboxylate transporter 1 inhibits the invasion and migration in human nasopharyngeal carcinoma cells [45]. Herein, we found unsurprisingly that knockdown of TP73-AS1 could inactivate this pathway in LAD cells and confirmed that TP73-AS1 play an oncogenic role in LAD through activating this pathway. Likewise, we revealed that TP73-AS1 had a regulatory effect on MEK/ERK pathway, which also belongs to EGFR signaling [46]. However, further study on this pathway was deficient here since it mainly plays a role in cell survival, proliferation, and differentiation [46], and we may put emphasis on it in the future.

All in all, our study verified the significant carcinogenic role of TP73-AS1 in the progression of LAD depending on PI3K/AKT signaling pathway, indicating TP73-AS1 as a novel prognostic biomarker and therapeutic target for LAD patients. However, our findings in the present study need to be further confirmed by experiments conducted in higher animals or even the clinical trials. Additionally, the detailed mechanism of TP73-AS1 modulating PI3K/AKT pathway in LAD is untouched here and this limitation will be broken in our future studies. Moreover, other regulatory mechanisms underlying TP73-AS1 affected LAD progression should also be explored in the future so as to justify further evaluation of TP73-AS1 as a potential therapeutic and prognostic target for patients with LAD.

## Supporting information

**Supplementary Figure S1:** 

**Supplementary Figure S2:** 

## Abbreviations

AKT (PKB): protein kinase B
AS1: antisense RNA 1
ATCC: American type culture collection
CCK8: cell counting kit 8
EGFR: epidermal growth factor receptor
EMT: epithelial–mesenchymal transition
ERK: extracellular signal-regulated kinases
GAPDH: glyceraldehyde 3-phosphate dehydrogenase
HCC: hepatocellular carcinoma
HMGB1: high mobility group box 1
HOTTIP: HOXA transcript at the distal tip
LAD: lung adenocarcinoma
lncRNA: long non-coding RNA
MEK: mitogen-activated protein kinase kinase
NC: nitrocellulose
NEAT1: nuclear enriched abundant transcript 1
NF-κB: nuclear factor kappa-light-chain-enhancer of activated B cells
NSCLC: non-small-cell lung cancer
OS: overall survival
PD-1/PD-L1: programmed cell death protein 1/programmed death-ligand 1
PFA: paraformaldehyde
PI3K: phosphoinositide 3-kinase
q-RT-PCR: quantification real-time polymerase chain reaction
RAGE: receptor for advanced glycation endproducts
RIPA: Radioimmunoprecipitation assay
RPMI 1640: Roswell Park Memorial Institute medium
TBST: TBS and Tween 20
TNM: tumor, node, metastasis
TP73: tumor protein P73
TLR4: toll-like receptor 4
