# Supplementary material for: LncRNA TP73-AS1 promoted the progression of lung adenocarcinoma via PI3K/AKT pathway
Source: Biosci Rep. 2019 Jan 11;39(1):BSR20180999. doi: 10.1042/BSR20180999 (PMC6328885; doi:10.1042/BSR20180999)
Supplement: Supplementary file 1 [file bsr20180999_Supp1.pdf]

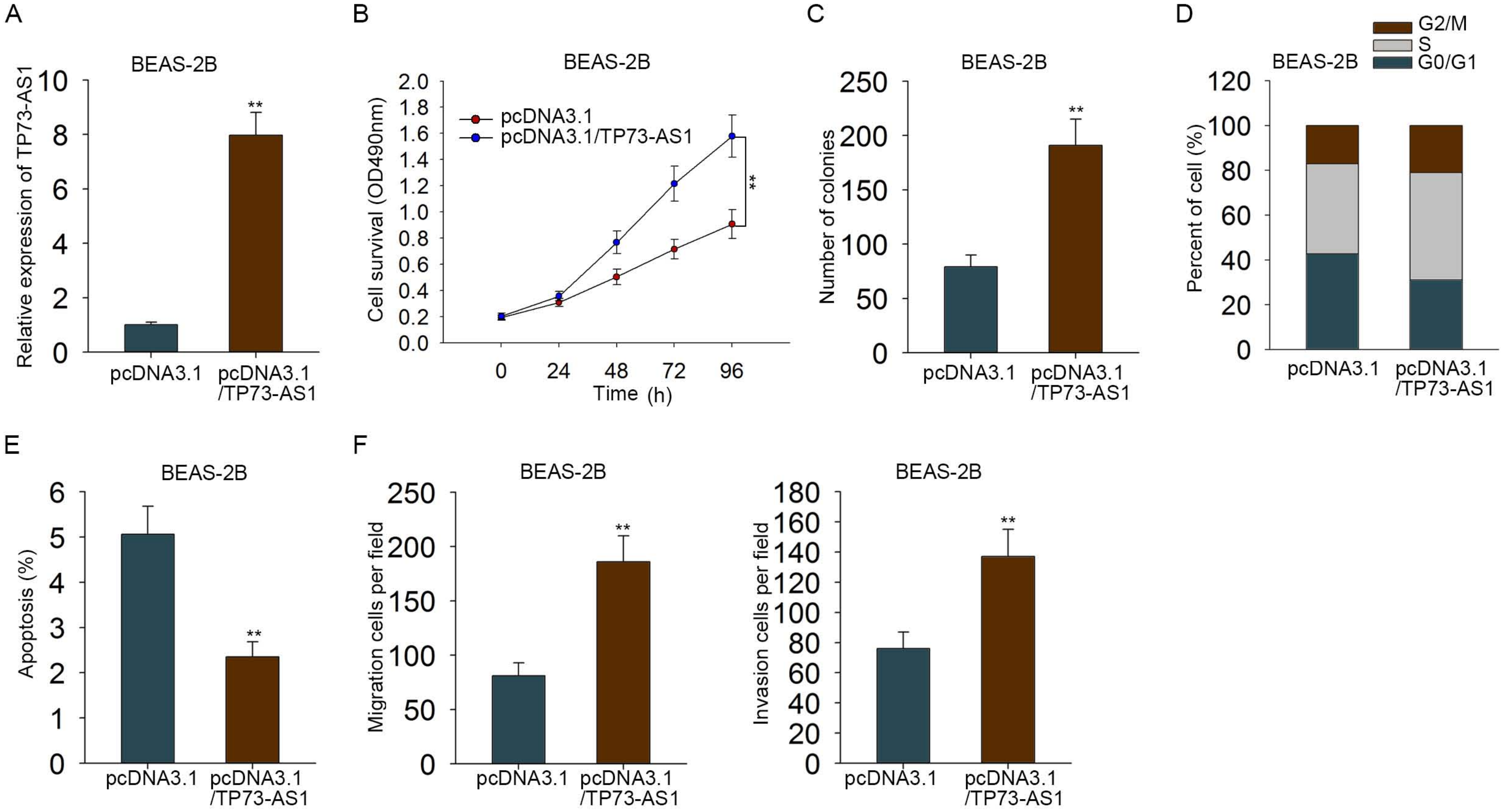
A
BEAS-2B
Relative expression of TP73-AS1
10
8
6
4
2
0
**
pcDNA3.1
pcDNA3.1
/TP73-AS1
B
BEAS-2B
Cell survival (OD490nm)
2.0
1.8
1.6
1.4
1.2
1.0
0.8
0.6
0.4
0.2
0.0
pcDNA3.1
pcDNA3.1/TP73-AS1
**
0
24
48
72
96
Time (h)
C
BEAS-2B
Number of colonies
250
200
150
100
50
0
**
pcDNA3.1
pcDNA3.1
/TP73-AS1
D
G2/M
S
G0/G1
BEAS-2B
Percent of cell (%)
120
100
80
60
40
20
0
pcDNA3.1
pcDNA3.1
/TP73-AS1
E
BEAS-2B
Apoptosis (%)
6
5
4
3
2
1
0
**
pcDNA3.1
pcDNA3.1
/TP73-AS1
F
BEAS-2B
Migration cells per field
250
200
150
100
50
0
**
pcDNA3.1
pcDNA3.1
/TP73-AS1
BEAS-2B
Invasion cells per field
180
160
140
120
100
80
60
40
20
0
**
pcDNA3.1
pcDNA3.1
/TP73-AS1

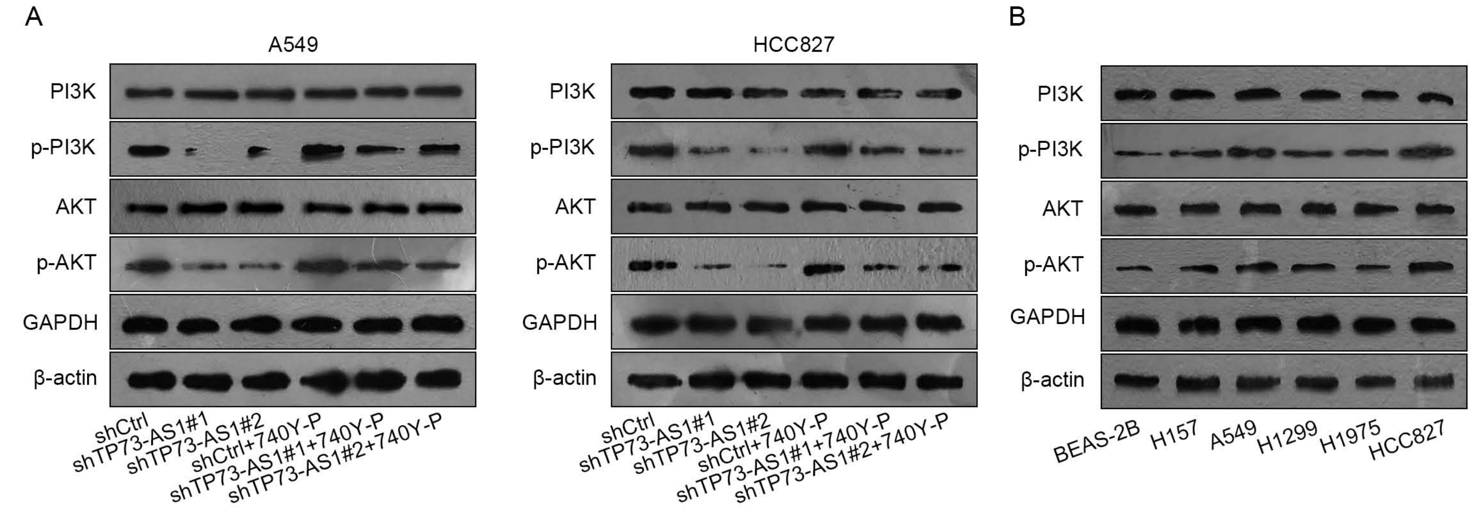
A
A549
HCC827
B
PI3K
p-PI3K
AKT
p-AKT
GAPDH
β-actin
shCtrl
shTP73-AS1#1
shTP73-AS1#2
shCtrl+740Y-P
shTP73-AS1#1+740Y-P
shTP73-AS1#2+740Y-P
BEAS-2B
H157
A549
H1299
H1975
HCC827
